# The Importance of the First 24 Postoperative Hours: Does Current Evidence Support Short-Stay High-Acuity Care After Gynecologic Oncology and Complex Abdominal Surgery?

**DOI:** 10.3390/jcm15166462

**Published:** 2026-08-20

**Authors:** Vasilios Pergialiotis, Maria Fanaki, Rafaela Panagopoulou, Pantelis Antonakis, Konstantinos Bramis, Emmanouil Stamatakis, Dimitrios Efthimios Vlachos, Dimitrios Haidopoulos, Nikolaos Thomakos

**Affiliations:** 1 First Department of Obstetrics and Gynecology, Division of Gynecologic Oncology, Alexandra Hospital, National and Kapodistrian University of Athens, Lourou Str., 11528 Athens, Greece; maria.fanaki@gmail.com (M.F.); rafaelapng@hotmail.com (R.P.); vlachos.dg@gmail.com (D.E.V.); dimitrioshaidopoulos@gmail.com (D.H.); thomakir@hotmail.com (N.T.); 2 Second Department of Surgery, Medical School, Aretaieio Athens Hospital, National and Kapodistrian University of Athens, 11528 Athens, Greece; pantonakis@med.uoa.gr (P.A.); kbramis@gmail.com (K.B.); 3 Department of Anesthesia, Alexandra Hospital, 11528 Athens, Greece; emmstamatakis@hotmail.com

**Keywords:** critical care, ICU, PACU, HDU, gynecologic oncology, abdominal surgery

## Abstract

**Background:** Postoperative admission to critical care facilities is frequently employed following complex abdominal and gynecologic oncology surgery; however, the clinical value of routine short-stay (≤24 h) high-acuity care remains uncertain as the existing evidence is limited, heterogeneous and derived primarily from non-randomized studies. Consequently, the present critical narrative review aims to discuss the rationale for planned short-duration postoperative high-acuity care, summarize the contemporary evidence, and identify priorities for future research. **Methods:** Relevant studies evaluating planned postoperative admission to critical care facilities for ≤24 h following major abdominal surgery were identified through a targeted review of contemporary literature using a structured search of MEDLINE, Scopus, Google Scholar, the Cochrane Central Register of Controlled Trials (CENTRAL) and ClinicalTrials.gov. Evidence from observational studies investigating intensive care units, high-dependency units, post-anesthesia care units, and advanced recovery pathways was critically synthesized, with particular attention to clinical outcomes, healthcare utilization, and current knowledge gaps. **Results:** Nine primary studies were included, predominantly observational in design, with the majority lacking a comparative study design, thereby limiting direct evaluation of the clinical effectiveness of planned short-stay postoperative high-acuity care. None of the studies were designed to evaluate a gynecologic oncology population, limiting the direct applicability of the available evidence to this setting. As such, the current evidence is better suited to critical clinical interpretation of the rationale, patient selection, and potential role of postoperative high-acuity care than to drawing definitive conclusions regarding intervention effectiveness. Overall, the available data suggest that planned short-stay high-acuity postoperative care is not consistently associated with reductions in postoperative mortality or overall morbidity but may decrease unplanned intensive care unit admissions in selected patient populations. Most studies reported little or no effect on overall hospital length of stay, whereas increased healthcare costs were primarily observed in capacity-driven or unplanned postoperative critical care pathways. Interpretation of the available literature remains limited by the predominance of observational studies, heterogeneous patient populations, variability in critical care models, and inconsistent outcome reporting. **Conclusions:** Current evidence does not support routine postoperative admission to critical care facilities following major abdominal surgery, while evidence specifically addressing gynecologic oncology remains insufficient to establish specialty-specific effectiveness. However, planned, time-limited high-acuity postoperative care was not associated with an observed increase in adverse clinical outcomes in the available observational studies and may offer clinical and system-level benefits in selected high-risk patients; these findings should be interpreted cautiously given the limitations of the available evidence. Prospective studies with standardized outcome reporting and clearly defined populations are needed to inform evidence-based perioperative care strategies.

## 1. Introduction

The incidence of gynecologic cancer has increased during the last decades, representing a substantial and growing global health burden. Collectively, the major gynecologic malignancies, including cervical, ovarian, endometrial, vulvar, and vaginal cancers account for more than 1.4 million new cases annually worldwide [1,2]. Despite advances in screening strategies and the introduction of novel systemic therapies, cancer-related mortality remains high as a significant proportion of patients continue to present with advanced-stage disease. In these cases, the need for multimodal treatment that involves high-complexity surgical procedures with multivisceral resections as well as adjuvant treatments results in substantial and prolonged physiological stress [3,4,5].

Parallel to this epidemiologic trend is the aging of the global cancer population. More than 60% of gynecologic cancers are now diagnosed in women aged ≥65 years in high-income regions, a proportion that steadily increases worldwide [6]. Elderly patients and particularly octogenarians often present with multiple comorbidities, frailty, and reduced physiological performance status, factors that significantly influence the perioperative risk, their functional recovery during the postoperative period, as well as their eligibility for aggressive oncologic treatment [7,8,9]. The interplay between advanced disease stage, complexity of surgical procedures, and patient comorbidities/performance status is of particular interest in modern perioperative planning, with optimization strategies aiming to provide the optimal level of care with the use of critical care facilities, which seem to help improve outcomes.

Critical care facilities have traditionally been utilized in gynecologic oncology to help counteract physiological postoperative stress response that may result in hemodynamic instability and transient organ dysfunction that can considerably increase the risk of developing postoperative complications. Modern management of surgical patients, including gynecologic cancer patients, involves preoperative strategies, under the scope of prehabilitation, that aim to improve patients’ physiology, thus reducing the risks of postoperative instability [10]. In the immediate postoperative period, proactive support of frail patients, as well as those submitted to high-complexity procedures, has been actively discussed in recent years [11,12]. In our recent review we highlighted the importance of instituting critical care to support complex oncologic surgery, as it seems that enhanced hemodynamic monitoring, early recognition of deterioration and optimization of pain control under a multidisciplinary team offers significant advantages to gynecologic cancer patients [13]. The optimal decision about the actual level of critical care support is not always easy, as several factors may result in a complex interplay, therefore rendering necessary a strategy tailored to each patient’s needs. In our era, several alternatives to the traditional intensive care unit (ICU) are available, including high-dependency units (HDU), post-anesthesia care unit (PACU) and advanced recovery room care (ARRC) designed to address different levels of patient need. While this structure seems particularly complex, it seems to be necessary as it aims to deliver intermediate levels of monitoring while mitigating the cost and capacity constraints associated with intensive care units (ICUs) [14,15].

Evidence specifically addressing the impact of critical care facilities in gynecologic oncology surgery remains limited and heterogeneous. Early observational studies demonstrated that postoperative ICU admission was common following major gynecologic cancer surgery and was largely driven by surgical complexity and extent of comorbidities, indicating the absence of a dedicated protocol for admission and/or discharge to the postoperative surgical unit [16,17]. In one of the earliest published studies, researchers supported that survival outcomes of high-risk gynecologic cancer patients admitted to an ICU following surgery were modest, as the reported overall mortality was 12%, which was at the time reported as acceptable [18]. In 2018, Ross et al. observed that unplanned admission to the intensive care unit, following cytoreductive surgery for ovarian cancer was an independent negative predictive factor of survival (27.3 vs. 57.9 months), indicating the need for proper management of patients to avoid destabilization and postoperative complications [19]. Predictors of ICU utilization in this study involved extensive cytoreduction, reduced performance status and significant intraoperative blood loss, providing a framework that could be considered for planned rather than reactive ICU admission.

Predictive factors of admission of gynecologic cancer patients to critical care units were first more than a decade ago by Ruskin et al. [17]. The authors observed that women age 60 years or older, those with hematocrit of 30% or less, albumin of 3.5 g/dL or less, as well as those with Charlson Comorbidity Index (CCI) score greater than 8, had a high risk of admission in an ICU. The authors concluded that admissions could be anticipated preoperatively, supporting the concept of selective, planned critical care use. At the same time we analyzed the indications of increased hospital stay in high dependency units (HDU) and observed that older age, the need for intraoperative invasive hemodynamic monitoring, bowel resection and intraoperative estimated blood loss were the predominant factors that prolonged hospitalization in HDU [20].

On the other hand, unplanned admissions to critical care facilities seem to be associated with prolonged hospitalization and decreased overall survival according to recent research [21]. This association indicates that the timing and intent of critical care admission may be as important as admission itself.

In summary, while critical care facilities play an increasingly important role in the management of abdominal surgery, and potentially gynecologic oncology as well, robust, disease-specific evidence remains limited, and consensus is still lacking on the actual importance of overnight admission immediately after the operation. To date, most of the available evidence is extrapolated from mixed populations of different medical specialties and some studies that refer to gastrointestinal surgery. In view of these considerations, the present narrative review aims to provide a comprehensive critical appraisal of the contemporary literature concerning planned short-duration (≤24 h) postoperative admission to critical care facilities following major abdominal surgery. Specifically, we sought to discuss the current rationale underpinning postoperative high-acuity care, synthesize the available evidence regarding its potential impact on clinically relevant outcomes and healthcare resource utilization, examine the factors that currently influence patient selection and postoperative triage, explore the applicability of the existing evidence to gynecologic oncology, and identify the principal methodological limitations and knowledge gaps that should inform future clinical research.

## 2. Methods

### 2.1. Literature Search and Scope

The present review provides a critical synthesis of the contemporary evidence regarding planned short-duration (≤24 h) postoperative admission to high-acuity care following major abdominal surgery, with particular emphasis on its potential role in gynecologic oncology. For the purposes of this review, “high-acuity care” is used as a general term to describe postoperative care environments that provide monitoring and support beyond standard ward-level care. However, these models should not be considered equivalent: intensive care units (ICUs), high-dependency units (HDUs), intermediate care units (IMCUs), post-anesthesia care units (PACUs), advanced recovery room care (ARRC), postoperative surgical units (POSUs), and post-anesthesia high-care units (PAHCUs) differ in staffing, monitoring intensity, admission criteria, and capacity for organ support. Accordingly, these distinctions were acknowledged when interpreting the available evidence, while the considerable variability among high-acuity care models was considered an important source of clinical and methodological heterogeneity.

Relevant publications were identified through targeted searches of the international literature using MEDLINE, Scopus, Google Scholar, the Cochrane Central Register of Controlled Trials (CENTRAL), and ClinicalTrials.gov, complemented by manual screening of reference lists of relevant articles. The review focuses on structured postoperative high-acuity care pathways, including intensive care units (ICUs), high-dependency units (HDUs), intermediate care units, post-anesthesia care units (PACUs), and advanced recovery models providing enhanced monitoring beyond standard ward care. Particular emphasis was placed on studies evaluating postoperative clinical outcomes, healthcare resource utilization, and perioperative management strategies, while evidence relating to prolonged critical care admission or capacity-driven postoperative boarding was considered only when relevant to the interpretation of current practice.

The literature search was conducted between 1 December 2025 and 13 January 2026, without restrictions on publication date. Searches combined terms related to postoperative care (“postoperative care,” “postoperative monitoring,” “postoperative recovery”), high-acuity or critical care (“critical care,” “intensive care,” “ICU,” “high-dependency unit,” “HDU,” “intermediate care,” “post-anesthesia care unit,” “PACU,” “advanced recovery room”), and the surgical population (“major abdominal surgery,” “abdominal surgery,” “non-cardiac surgery,” “gynecologic oncology,” “gynecologic cancer”). Search syntax was adapted to the requirements of each database. The complete search strategies are provided in Supplementary Table S1. Reference lists of included studies and relevant review articles were also screened to identify additional potentially eligible publications. No publication-date restrictions were applied. The date of final search was set to 30 May 2026. Articles written in Latin were considered eligible for retrieval and evaluation. Titles and abstracts of potentially relevant records were screened independently by two reviewers (R.P. and M.F.) according to the eligibility criteria described below. Potentially eligible articles were subsequently evaluated in full text by the same reviewers. Disagreements regarding eligibility were resolved through discussion and, when necessary, consultation with a third reviewer (V.P.).

Eligible interventions included planned postoperative admission to critical care facilities for a short duration (≤24 h) following major abdominal surgery. Critical care facilities are defined as intensive care units (ICU), high-dependency units (HDU), intermediate care units, or structured high-acuity postoperative care models (e.g., PACU-based advanced recovery or equivalent), providing enhanced monitoring and organ support beyond standard ward-level care. Both elective and unplanned admissions were considered, provided the intended or actual duration of critical care stay did not exceed 24 h. Studies in which short-stay critical care is delivered as part of a protocolized postoperative pathway or compared with standard ward-based care were considered eligible. Studies focusing exclusively on prolonged (>24 h) critical care admission without separable short-stay data were excluded. Therefore, interventions involving long-term mechanical ventilation, extracorporeal organ support, or salvage critical care initiated in response to established postoperative organ failure were excluded where these represent primary indications for admission rather than planned short-stay monitoring. Unplanned escalation to high-acuity care following perioperative deterioration and capacity-driven overnight postoperative boarding were considered separately from planned, protocolized admission and were retained only when they provided relevant information regarding postoperative triage, clinical outcomes, healthcare resource utilization, length of stay, or costs. While we aimed to primarily evaluate differences among patients admitted to critical care units for a short postoperative stay (<24 h) and those hospitalized in the postoperative surgical unit (POSU), we also chose to include non-comparative studies. Therefore, all randomized controlled trials as well as observational (prospective or retrospective) case–control/cohort studies were considered for inclusion in the present review. Case reports, experimental studies and conference proceedings were excluded. Review articles and other secondary sources were used to provide contextual information and support the broader narrative discussion but were not considered part of this primary evidence set.

**Figure 1:**
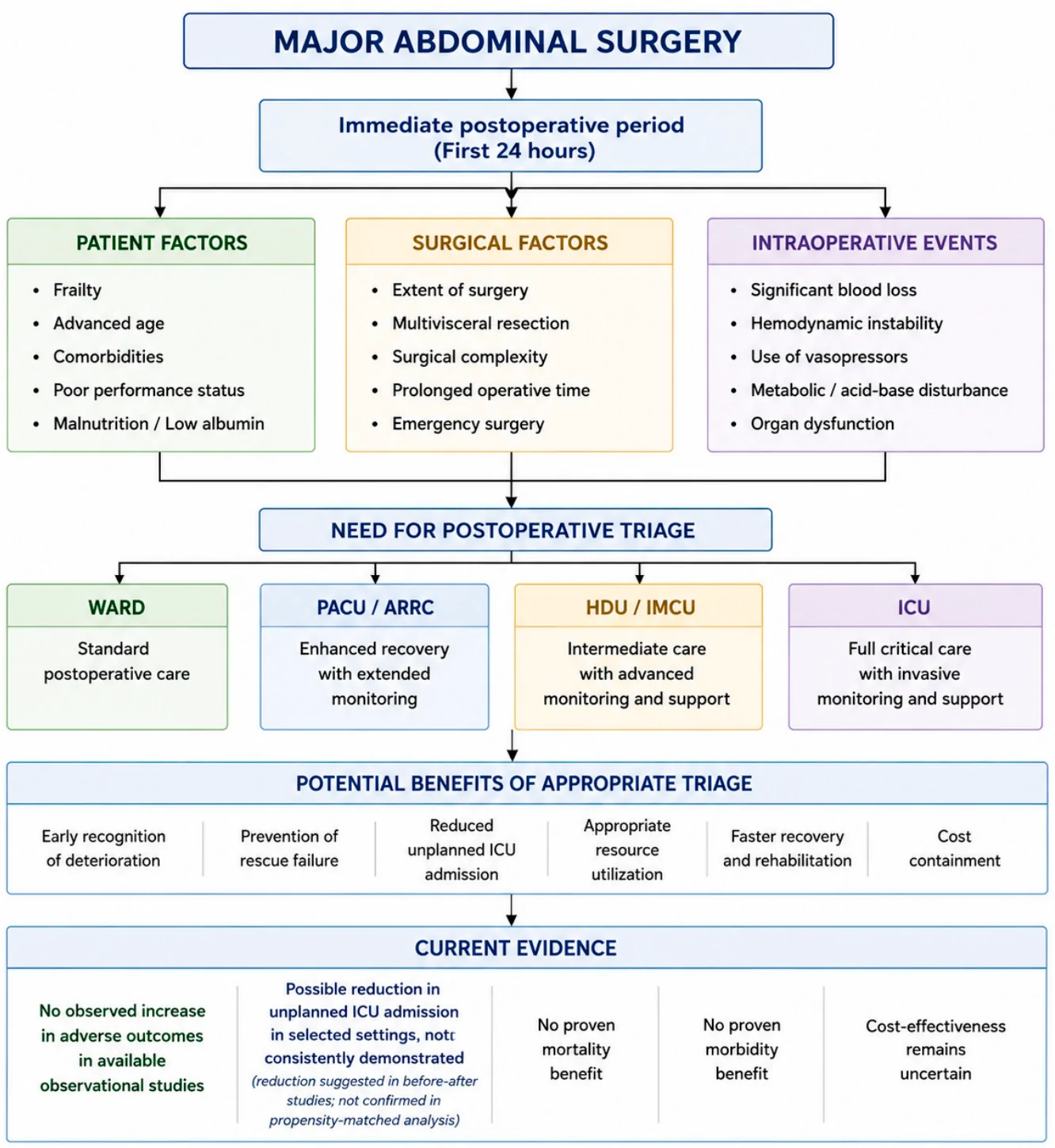
Conceptual model of postoperative triage during the first 24 h after major abdominal surgery. Patient-related factors, surgical complexity, and intraoperative physiological events collectively determine the need for postoperative monitoring and allocation to different levels of high-acuity care. This model served as the conceptual basis for the organization and interpretation of the available evidence presented in this review.

The thematic approach adopted in the present review, which guided the organization and interpretation of the available evidence, is summarized in Figure 1. The review focuses on the first 24 postoperative hours as the critical period during which patient characteristics, surgical complexity, and intraoperative physiological events collectively determine the need for postoperative monitoring and the appropriate level of high-acuity care. During this early postoperative window, physiological instability, occult complications, and deviations from the expected recovery trajectory are most likely to become clinically apparent, making timely recognition and appropriate escalation of care particularly important. Rather than considering postoperative critical care as a single intervention, the conceptual framework recognizes it as a continuum of monitoring strategies ranging from standard ward care to intermediate and intensive care environments, with allocation primarily driven by individualized patient risk and perioperative physiological requirements. Consequently, the review is organized around the interaction between these three principal domains, namely, (i) patient-related factors, (ii) procedure-related characteristics, and (iii) intraoperative events, and their potential influence on postoperative triage decisions, clinical outcomes, and healthcare resource utilization. This approach also provides a common framework for interpreting studies that differ substantially in patient populations, models of postoperative care, and reported outcome measures, facilitating comparison across the available literature despite the considerable methodological heterogeneity that characterizes this field. The conceptual framework was therefore used to organize the synthesis of the available evidence presented in the following sections and to identify the principal areas in which current knowledge is sufficient, where uncertainty persists, and where future research efforts should be directed.

### 2.2. Synthesis of the Evidence

The available literature was critically reviewed to summarize current knowledge regarding the role of short-duration postoperative critical care after major abdominal surgery. Evidence from randomized and observational studies was interpreted in the context of differences in patient populations, surgical complexity, perioperative pathways, and models of postoperative care. Particular attention was given to postoperative morbidity, mortality, unplanned escalation of care, hospital length of stay, healthcare costs, and findings applicable to gynecologic oncology. Owing to the considerable heterogeneity in study design, reported outcomes, and definitions of postoperative high-acuity care, findings were synthesized qualitatively to identify consistent evidence, areas of uncertainty, and priorities for future research. As this review is based exclusively on previously published literature, institutional review board approval and informed consent were not required.

While related outcomes and outcome measures were not predefined during the design of this review, we primarily sought to evaluate differences in postoperative outcomes among patients admitted to a critical care facility for a short-stay, including postoperative complications and length of stay. Differences in the economic burden of a short clinical stay were also considered. Evidence referring specifically to gynecologic cancer surgery was evaluated when available.

Methodological quality of the primary studies was critically appraised using design-specific Joanna Briggs Institute (JBI) critical appraisal tools. Comparative cohort studies were assessed using the JBI checklist for cohort studies [22], before–after or interrupted time-series evaluations using the JBI tool for quasi-experimental studies [23], and uncontrolled descriptive observational studies using the JBI checklist for case series [24]. Methodological quality appraisal was performed independently by two reviewers (M.F. and D.E.V.) using the JBI instrument appropriate to each study design. Disagreements regarding individual domain assessments were resolved through discussion and consensus, with consultation of a third reviewer (V.P.) when required. An explanation of the individual JBI domains assessed for each study design is provided in the Supplementary Materials.

## 3. Results

We identified nine studies evaluating planned short-duration (≤24 h) postoperative admission to high-acuity care following major abdominal surgery, representing the current body of published evidence on this topic [14,25,26,27,28,29,30,31,32]. None of the nine primary studies was specifically designed to evaluate a gynecologic oncology population, although gynecologic surgical patients were represented within some broader surgical cohorts [25,27] without separate reporting of gynecologic oncology-specific outcomes. Accordingly, the primary evidence set was derived predominantly from broader abdominal and non-cardiac surgical populations; therefore, findings from these studies should not be interpreted as direct evidence of effectiveness in gynecologic oncology. These studies encompassed a broad range of postoperative care models, including intensive care units (ICUs), high-dependency units (HDUs), post-anesthesia care units (PACUs), intermediate care units, and advanced recovery pathways, although relatively few specifically addressed gynecologic oncology populations. The methodological characteristics of these representative studies are summarized in Table 1. Most investigations were retrospective observational analyses, service evaluations, or feasibility studies, while randomized evidence remains unavailable. Several studies evaluated protocolized postoperative high-acuity pathways, whereas others described institution-specific or capacity-driven models of care. Table 2 summarizes the appraisal of the methodological quality of included studies and indicates substantial heterogeneity in the quality and strength of the available evidence. While several studies were methodologically adequate for describing postoperative high-acuity care populations and clinical pathways, the certainty regarding comparative effectiveness was limited by non-randomized designs, absence of appropriate comparator groups, and residual confounding. Even among the stronger comparative studies, potential selection and confounding biases remained, indicating that the current evidence should primarily be considered hypothesis-generating rather than sufficient to establish a causal benefit of planned short-duration high-acuity care. Consequently, interpretation of the available evidence should acknowledge the inherent limitations associated with observational study designs, including residual confounding, heterogeneous patient populations, and variation in perioperative management strategies.

Considerable variability was also observed in the outcomes reported across the available studies (Table 3). Mortality, unplanned escalation to critical care, and duration of postoperative high-acuity care were the most consistently evaluated endpoints, reflecting the traditional emphasis of perioperative critical care research on patient safety and healthcare resource utilization. In contrast, postoperative organ-specific complications, ward-level deterioration, patient-reported outcomes, quality of recovery, and cost-effectiveness were reported inconsistently or omitted altogether. Even when similar clinical outcomes were assessed, important differences existed in their definitions, measurement methods, follow-up periods, and reporting metrics, making direct comparisons across studies challenging. This variability likely reflects differences in institutional priorities, local postoperative care pathways, and the absence of standardized outcome sets for studies evaluating postoperative high-acuity care. Consequently, although the existing literature provides valuable insights into the potential role of structured postoperative monitoring during the first 24 h after surgery, the heterogeneity of reported outcomes limits robust comparisons between different models of care and hinders the identification of patient populations most likely to benefit. These observations further emphasize the need for future prospective studies adopting standardized definitions and core outcome measures to facilitate meaningful comparisons and strengthen the evidence base.

### 3.1. Morbidity and Mortality

Morbidity and mortality outcomes were reported in 4 studies [14,25,27,28]. Specifically, Koning et al. reported the absence of differences in postoperative 30-day (2.8 for IMCU vs. 2.7 for PACU), 3-month (5.9% for IMCU vs. 5.1 for PACU) and 1-year (10.8% for IMCU vs. 8.9% for PACU) mortality rates as well as in postoperative complications (8.5% for IMCU vs. 9.6% for PACU). Costa-Pinto reported an in-hospital mortality rate of 2.9% (37/1257) among patients admitted to the recovery high-dependency unit; 13.0% (164/1257) subsequently required intensive care unit admission, while 6.4% (70/1093) of patients discharged to the ward experienced a medical emergency team call within 24 h. However, significant differences existed among baseline patient characteristics, including comorbidities (evaluated with the Charlson index) and overall performance status (evaluated with APACHE-3 score), thereby making the interpretation of these findings difficult. However, they observed that the actual impact of an overnight stay at the ICU was associated with relatively low rates (6.4% (70/1093)) of a medical emergency team call within 24 h of discharge from the critical care unit. Ludbrook et al. indicated in their first pilot study that the introduction of an advanced recovery room is feasible [27], but in their complete report 3 years later, they did not detect significant differences in terms of 30-day (1.4% vs. 2.6%; odds ratio [OR] 0.5, 95% confidence interval [CI] 0.2–1.6; *p* = 0.42) or 90-day (3.2% vs. 5.4%; OR 0.3, 95% CI 0.1–1.0; *p* = 0.25) mortality. Medical emergencies were more frequent among patients admitted in the critical care facility for an overnight stay (*p* < 0.001), an effect that seemed to be independent of the actual patient characteristics as these were well balanced [14].

### 3.2. Admission to ICU and Length of Stay

Despite broad agreement that postoperative triage should incorporate patient-related risk, procedural complexity, and perioperative physiological requirements, the available literature demonstrates considerable variability in how these factors were translated into admission decisions. Formal risk stratification tools were incorporated inconsistently. The American College of Surgeons National Surgical Quality Improvement Program (ACS NSQIP) Surgical Risk Calculator was explicitly used by Ludbrook et al. to define medium-risk surgical populations considered for advanced recovery room care (ARRC) [14], whereas Harmse et al. reported that the American Society of Anesthesiologists (ASA) Physical Status Classification, Lee’s Revised Cardiac Risk Index (RCRI), frailty assessment, the STOP-Bang questionnaire, and the ACS NSQIP Surgical Risk Calculator informed preoperative assessment, although no fixed admission algorithm was applied [26]. Most other studies relied predominantly on institution-specific admission criteria and multidisciplinary clinical judgement, frequently incorporating anticipated monitoring requirements, comorbidity burden, surgical complexity, or expected cardiovascular and respiratory support requirements. Koning et al., for example, directed high-risk patients with an anticipated uncomplicated recovery within 24 h to the post-anesthesia care unit (PACU), whereas an anticipated need for prolonged critical care prompted direct admission to the intensive care unit (ICU) at the discretion of the treating anesthesiologist [25].

Unplanned ICU admission and escalation of care were among the most frequently reported outcomes. Specifically, Koning et al. reported no difference in the overall rate of postoperative ICU admission between the IMCU and PACU periods (4.0% vs. 4.1%, respectively; *p* = 0.929), while ICU admission with a strict medical indication was numerically lower during the PACU period (3.8% vs. 2.6%; *p* = 0.062), although this difference did not reach statistical significance [25]. Readmission rates also significantly decreased in this group (12.1% vs. 14.7% *p* = 0.032). Similarly, Ludbrook et al. observed that the actual unplanned ICU admissions were significantly reduced following the introduction of dedicated overnight stay in an advanced recovery room care (ARRC) in their pilot study [27]. In the subsequent propensity score–matched study, ICU admission did not differ significantly between patients receiving ARRC and usual care (4.3% vs. 2.9%; odds ratio 1.5, 95% confidence interval 0.7–3.4; *p* = 0.42) [14]. Three additional non-comparative studies reported widely varying rates of ICU admission (1.07–13% of cases), without indicating the actual reason [26,28,29].

In terms of length of hospitalization, most of the studies that evaluated this outcome reported no meaningful differences among patients that were admitted in a critical care facility for less than 24 h compared to that of patients directly transferred to POSU following the operation [14,25,27,32].

### 3.3. Healthcare Resource Utilization and Economic Impact

Only one study evaluated the healthcare resource utilization of an overnight stay in critical care facilities following abdominal surgery [32]. The authors observed increased hospital costs associated with capacity-driven overnight PACU boarding. Specifically, among patients with an expected resource length of stay ≥7 days, overnight PACU boarding was associated with an excess stay of 1.066 days per patient (95% confidence interval [CI] 0.047–2.022), corresponding to 138.69 excess bed-days (95% CI 6.12–262.91) over the study period. In contrast, no increase was observed among patients with an expected stay of 2 to <7 days (−0.004 days per patient, 95% CI −0.132 to 0.099). Overnight PACU status was not significantly associated with mortality (*p* = 0.445).

## 4. Discussion

The findings of the present review suggest that planned, protocolized high-acuity postoperative care was not associated with an observed increase in postoperative morbidity or mortality in the available observational studies and may offer potential benefits in selected settings, although reductions in unplanned ICU admission have not been consistently demonstrated. However, interpretation of these findings requires consideration of the distinct postoperative pathways represented in the available literature. Planned high-acuity care, including structured or protocolized pathways, should be distinguished from unplanned escalation to critical care following postoperative deterioration and from capacity-driven overnight boarding resulting from limitations in postoperative bed availability. These categories represent clinically different exposures and are subject to different indications and sources of confounding. Importantly, evidence indicating increased resource utilization and prolonged hospitalization was derived from capacity-driven overnight PACU boarding and should therefore not be interpreted as evidence of adverse economic consequences of planned postoperative high-acuity care. Similarly, outcomes associated with unplanned escalation reflect deterioration requiring a higher level of care rather than the effect of a planned postoperative monitoring strategy. Viewed through the conceptual model proposed in Figure 1, these findings indicate that postoperative high-acuity care should not be regarded as a universal intervention but rather as a structured triage strategy during the first 24 postoperative hours, when patient-related characteristics, surgical complexity, and intraoperative physiological disturbances interact to determine the need for enhanced monitoring and timely escalation or de-escalation of care. Within this context, the principal clinical question is unlikely to be whether postoperative critical care is universally beneficial, but rather which patients are most likely to derive meaningful benefit from protocolized short-duration monitoring. This question remains difficult to address due to the considerable heterogeneity observed through the available literature. Mortality, unplanned ICU admission, and duration of postoperative critical-care stay were the most consistently reported endpoints, whereas organ-specific complications, ward-level deterioration, patient-reported recovery, and health-economic outcomes were evaluated inconsistently or omitted altogether. Even when similar outcomes were assessed, important differences existed in their definitions, measurement methods, and follow-up periods, limiting direct comparisons between studies. Furthermore, heterogeneous patient selection, variation in perioperative care pathways, and residual confounding preclude definitive causal conclusions. Consequently, although the available evidence supports consideration of individualized, risk-based postoperative care allocation, it remains insufficient to define specific criteria for escalation or de-escalation or to determine the optimal level of monitoring for individual patients.

Direct evidence evaluating planned short-duration postoperative high-acuity care specifically in gynecologic oncology remains scarce. Accordingly, the findings derived from broader abdominal and non-cardiac surgical populations cannot establish effectiveness in gynecologic oncology and should be regarded as hypothesis-generating when applied to this population. Nevertheless, the characteristics of patients undergoing complex gynecologic oncology surgery provide a clinically relevant rationale for investigating this strategy prospectively. In our previous article [13] we emphasized the need for structured perioperative monitoring and judicious allocation of high-acuity care and the present review serves as the basis for the conduct of future studies denoting the actual gaps in current research. Since a substantial number of gynecologic cancer patients are expected to be admitted to a critical care facility, it is important to evaluate at which timepoint it is best to introduce critical care following surgery.

Major complications arise in a substantial number of gynecologic cancer patients submitted to complex gynecologic oncology surgery as landmark studies indicate that severe morbidity may affect up to 22% of patients [33,34,35], with newer cohorts reporting rates approaching 50% [36]. Accumulating evidence suggests that most complications arise during the early postoperative period, particularly within the first 24–48 h [37,38]. This observation underlines the necessity of intensive early monitoring of these patients as prevention or at least control of their extent may exert a significant effect on both short-term recovery and longer-term oncologic outcomes.

Moreover, advances in perioperative management, and postoperative care have progressively expanded surgical eligibility, in a way that allows patients with reduced functional reserve who were previously deemed unsuitable for major oncologic surgery to be offered standard and even extensive surgical procedures, albeit with a heightened risk of postoperative morbidity [39,40]. Deterioration of frail patients may occur rapidly, resulting in multiorgan dysfunction that significantly prolongs the need for hospitalization and increases the possibility of long-term deficits [41,42]. It becomes important to understand if this group of patients would benefit from an early, triage-based admission to a critical care facility for early postoperative recovery and follow-up. To date, however, relevant evidence is lacking, leading to significant discrepancies in patient care that are primarily capacity- and facility-driven.

It is also worth mentioning that the relationship between ERAS pathways and postoperative high-acuity care also warrants consideration. High-acuity care represents a decision regarding the intensity and location of postoperative monitoring rather than an alternative perioperative recovery pathway and may therefore coexist with ERAS principles when clinically indicated. However, the included studies did not specifically evaluate the interaction between ERAS implementation and short-duration high-acuity care, precluding conclusions regarding their combined impact on postoperative outcomes.

## 5. Strengths and Limitations

Unlike previous publications focusing primarily on individual critical care models or specific surgical specialties, the present review integrates evidence across different postoperative monitoring environments while specifically focusing on the first 24 postoperative hours, which is the period during which postoperative triage decisions are most likely to influence clinical outcomes. By synthesizing evidence from intensive care units (ICUs), high-dependency units (HDUs), post-anesthesia care units (PACUs), and advanced recovery pathways within a clinically relevant risk stratification model, the review provides a contemporary and unified perspective on postoperative monitoring following major abdominal surgery, with particular emphasis on its application to gynecologic oncology. In doing so, it highlights both areas of consensus and the persistent uncertainties that continue to limit evidence-based decision-making in this evolving field.

Nevertheless, several limitations should be acknowledged. First, the available evidence remains limited and is characterized by considerable heterogeneity in study design, patient populations, definitions of postoperative high-acuity care, and reported outcome measures. The limited comparative evidence and heterogeneity of the evaluated populations and care pathways therefore preclude conclusions regarding whether escalation or de-escalation between different levels of postoperative care, or selection of a particular high-acuity care model, is associated with improved clinical outcomes. Furthermore, all available studies are observational, with no randomized controlled trials currently available, limiting the ability to draw definitive conclusions regarding the superiority, or lack thereof, of planned short-duration postoperative critical care compared with conventional ward-based management. In addition, none of the nine primary studies was specifically designed to evaluate a gynecologic oncology population, and evidence specific to this population therefore remains scarce, necessitating cautious extrapolation from broader abdominal and non-cardiac surgical populations and interpretation of the resulting gynecologic oncology implications as largely extrapolative and hypothesis-generating. Substantial variability in outcome selection, outcome definitions, and reporting methods limits comparisons across studies and highlights the need for standardized core outcome sets in future research. Finally, methodological appraisal using design-specific Joanna Briggs Institute critical appraisal tools demonstrated variable methodological quality across the included studies. Although several studies were adequate for describing postoperative high-acuity care populations and clinical pathways, the evidence regarding comparative effectiveness remains limited by the predominance of non-randomized designs, the absence of appropriate comparator groups in several studies, and potential selection bias and residual confounding even among comparative studies. Accordingly, the available evidence should primarily be regarded as hypothesis-generating, and definitive causal conclusions regarding the benefit of planned short-duration postoperative high-acuity care cannot presently be drawn.

### Implications for Future Research and Clinical Practice

The findings of the present review suggest that current evidence does not support routine postoperative admission to a critical care facility for all patients undergoing major abdominal or gynecologic oncology surgery. Nevertheless, planned short-stay admission to high-acuity care may remain appropriate in selected patients, particularly when driven by decisions related to preoperative baseline characteristics and intraoperative physiological responses that denote a potentially high-risk immediate postoperative period. It is beyond the scope of the present review to definitively suggest that critical care facilities are without any benefit in selected cases and factors such as impaired performance status, advanced age or frailty, significant comorbidity burden, and intraoperative features including hemodynamic instability, metabolic derangements, substantial blood loss, or need for vasoactive support should be systematically evaluated when determining postoperative care level. In this context, structured and protocolized triage decisions may offer a more rational alternative to indiscriminate ICU admission or capacity-driven practices.

There is a clear need for prospective clinical trials designed to address the limitations of the existing observational evidence. Such studies should apply strict and transparent population selection criteria, ideally distinguishing between cohorts defined by patient vulnerability (e.g., frailty or advanced age) and those characterized by surgical complexity (e.g., extensive cytoreductive or multivisceral procedures) to help limit confounding bias. Moreover, trials should incorporate predefined and comprehensive outcome sets that capture not only mortality and ICU utilization but also postoperative morbidity, adverse ward-level events, patient-centered outcomes, and cost-effectiveness, thereby minimizing selective reporting and enabling robust evaluation of benefit.

## 6. Conclusions

This study highlights the limited and heterogeneous nature of the current evidence evaluating planned short-stay (≤24 h) postoperative admission to critical care facilities following major abdominal surgery. Overall, routine admission to high-acuity care cannot be supported based on available data; however, the available observational evidence did not demonstrate an observed increase in adverse clinical outcomes associated with selective use of structured, time-limited postoperative monitoring, while potential benefits in appropriately selected patients remain to be established prospectively. Importantly, evidence specifically addressing gynecologic oncology remains scarce, and findings derived from broader abdominal and non-cardiac surgical populations should be considered hypothesis-generating. It should be noted that most of the evidence referring to the negative impact of routine admission to critical care facilities, in terms of length of stay and system costs, are obtained from studies evaluating capacity-driven or unplanned practices rather than intentional care pathways. Therefore, these should be interpreted separately and cannot be used to infer adverse consequences of planned postoperative high-acuity care. Moreover, interpretation of findings is constrained by the observational design of included studies, inconsistent outcome reporting, and persistent concerns regarding selection bias and residual confounding, which collectively limit the methodological robustness of the available evidence and preclude definitive conclusions. Moreover, none of the nine primary studies was specifically designed to evaluate a gynecologic oncology population, limiting the direct applicability of the available evidence to this setting. This is why future research should focus on well-designed prospective trials with clearly defined populations, standardized outcome reporting, and attention to high-risk subgroups such as frail or elderly patients and those undergoing complex procedures, to better inform evidence-based perioperative care strategies.

## Figures and Tables

**Table 1:** Methodological characteristics of included studies. Summary of the design features and populations of studies investigating short-duration postoperative critical-care pathways, including PACU, ARRC, IMC/IMCU, POSU, PAHCU, and short-stay HDU models. Only studies in which the intervention involved a monitored postoperative stay of ≤24 h were included. Comparator arms may include units with longer stays (e.g., IMCU) where the intervention arm met short-stay criteria. Abbreviations: ARRC, Advanced Recovery Room Care; PACU, Post-Anesthesia Care Unit; HDU, High Dependency Unit; IMC/IMCU, Intermediate Care/Intermediate Care Unit; POSU, Postoperative Surgical Unit; PAHCU, Perioperative Adult High-Care Unit; LOS, length of stay.

Year; Author	Design	Population	Intervention	Comparator	Patients
2024; Koning [25]	Retrospective Before–After	All adult noncardiac surgical patients before/after a system-level change	PACU short-stay high-acuity care (<24 h)	IMCU (longer stay, pre-change model)	1554
2023; Harmse [26]	Retrospective Single-arm	Adults admitted to a perioperative high-care unit	PAHCU monitored high-acuity care stay, majority <24 h	None	1020
2023; Ludbrook [14]	Prospective observational with propensity matching	Moderate-risk adults undergoing elective/unplanned noncardiac surgery (predicted mortality 0.7–5%)	ARRC extended recovery monitored stay ≤24 h	Standard PACU → ward care	871 (ARRC 452; Ward 419)
2023; Costa-Pinto [28]	Retrospective Single-arm	Adult surgical patients admitted postoperatively to a recovery HDU	Overnight recovery HDU monitored stay, median ≈12 h	None	1257
2021; Ludbrook [27]	Prospective Before–After	Adults ≥18, moderate-risk noncardiac/non-neurosurgical surgery; predicted mortality 1–4%	ARRC monitored care ≤24 h	Pre-ARRC usual care	166 (Before 95; After 71)
2020; Nelson [32]	Retrospective Unplanned PACU overnight vs. ward	Adults ≥18 undergoing surgery with postoperative PACU stay	Overnight PACU stay ≤24 h with continued monitoring	No PACU overnight stay (ward or other unit)	17,466 (778 vs. 16,688)
2020; van Tunen [29]	Retrospective Single-arm	All adult PACU/IMC postoperative patients	PACU/IMC short-stay (<24 h)	None	>12,000
2014; Ganter [30]	Prospective Single arm (two PACU intensity levels)	Adult PACU patients (standard vs. IMC-level PACU)	Standard PACU LOS ~3 h; IMC-PACU LOS ~15 h → all <24 h	None	12,179
2008; Heller [31]	Prospective Before–After (Service Evaluation)	Adult patients undergoing major elective surgery	POSU postoperative surgical unit, ~24 h level-2 care	Pre-POSU era	1380 (Before 503; After 877)

**Table 2:** Methodological quality assessment of included studies. Critical appraisal of the primary studies using design-specific Joanna Briggs Institute (JBI) critical appraisal checklists. Studies were assessed using the JBI instrument considered appropriate for their respective study design, including the JBI Checklist for Cohort Studies, JBI Checklist for Quasi-Experimental Studies, and JBI Checklist for Case Series. Individual methodological criteria (Q1–Q11, as applicable to each instrument) were classified as Yes (green), No (red), Unclear (yellow), or Not applicable (N/A). An explanation for each domain assessed per question, per study design is provided in the Supplementary Materials.

**Cohort studies**											
**Study**	**Q1**	**Q2**	**Q3**	**Q4**	**Q5**	**Q6**	**Q7**	**Q8**	**Q9**	**Q10**	**Q11**
Ludbrook, 2023 [14]	🟢	🟢	🟢	🟢	🟢	🟢	🟢	🟢	🟡	🟡	🟢
Nelson, 2020 [32]	🟢	🟢	🟢	🟢	🟢	🟢	🟢	🟢	🟢	N/A	🟢
van Tunen, 2020 [29]	🔴	🟢	🟢	🟢	🔴	🟢	🟢	🟢	🟢	N/A	🟢
**Quasi-experimental studies**											
**Study**	**Q1**	**Q2**	**Q3**	**Q4**	**Q5**	**Q6**	**Q7**	**Q8**	**Q9**		
Koning, 2024 [25]	🟢	🟢	🟡	🟢	🟢	🟡	🟢	🟢	🟢		
Ludbrook, 2021 [27]	🟢	🟢	🟡	🟢	🔴	🟢	🟢	🟢	🟢		
Heller, 2008 [31]	🟢	🟡	🟡	🟢	🔴	🟡	🟡	🟡	🔴		
**Case series studies**											
**Study**	**Q1**	**Q2**	**Q3**	**Q4**	**Q5**	**Q6**	**Q7**	**Q8**	**Q9**	**Q10**	
Harmse, 2023 [26]	🟢	🟢	🟢	🟢	🟢	🟢	🟢	🟢	🟢	🟢	
Costa-Pinto, 2023 [28]	🟢	🟢	🟢	🟢	🟡	🟢	🟢	🟢	🟢	🟢	
Ganter, 2014 [30]	🟡	🟢	🟢	🟢	🟡	🔴	🟢	🟢	🟢	🟢	

**Table 3:** Outcome reporting across studies evaluating short-stay (≤24 h) postoperative critical-care models. Outcomes are grouped into domains of mortality, general and system-specific complications, respiratory and cardiovascular events, escalation of care (unplanned ICU admission), ward-based deterioration (MET/MER events), readmission, hospital length of stay, and duration of critical-care stay. **A:** formally analyzed outcome; **D:** outcome reported descriptively without formal comparative effectiveness analysis; **NR:** outcome not reported.

	Mortality	Complications	Respiratory	Cardiovascular	Unplanned ICU	Ward Adverse Events	Readmission	Hospital LOS	Critical-care LOS
Koning 2024 [25]	**A**	**A**	**A**	**A**	**A**	**NR**	**A**	**A**	**A**
Ludbrook 2023 [14]	**A**	**A**	**D**	**D**	**A**	**A**	**A**	**A**	**D**
Harmse 2023 [26]	**D**	**D**	**NR**	**NR**	**D**	**NR**	**NR**	**D**	**A**
Costa-Pinto 2023 [28]	**D**	**D**	**NR**	**NR**	**D**	**D**	**NR**	**D**	**D**
Ludbrook 2021 [27]	**D**	**D**	**D**	**D**	**D**	**D**	**D**	**D**	**D**
Nelson 2020 [32]	**A**	**NR**	**NR**	**NR**	**NR**	**NR**	**NR**	**A**	**D**
van Tunen 2020 [29]	**A**	**D**	**NR**	**NR**	**A**	**NR**	**A**	**NR**	**D**
Ganter 2014 [30]	**NR**	**D**	**NR**	**NR**	**NR**	**NR**	**NR**	**NR**	**A**
Heller 2008 [31]	**D**	**NR**	**NR**	**NR**	**D**	**NR**	**NR**	**NR**	**D**

## Data Availability

No new data were created or analyzed in this study. Data sharing is not applicable to this article.

## Supplementary Materials

The following supporting information can be downloaded at: https://www.mdpi.com/article/10.3390/jcm15166462/s1.
