# Supplementary material for: The Importance of the First 24 Postoperative Hours: Does Current Evidence Support Short-Stay High-Acuity Care After Gynecologic Oncology and Complex Abdominal Surgery?
Source: J Clin Med. 2026 Aug 20;15(16):6462. doi: 10.3390/jcm15166462 (PMC13514232; doi:10.3390/jcm15166462)
Supplement: Supplementary file 1 [file jcm-15-06462-s001.zip › jcm-4466012-supplementary.pdf]

Supplementary

Source	Proposed search strategy
**MEDLINE**	("Postoperative Care"[Mesh] OR postoperative care OR postoperative monitoring OR postoperative recovery) AND ("Critical Care"[Mesh] OR "Intensive Care Units"[Mesh] OR critical care OR intensive care OR ICU OR high-dependency unit OR HDU OR intermediate care OR post-anesthesia care unit OR PACU OR advanced recovery room) AND (major abdominal surgery OR abdominal surgery OR noncardiac surgery OR non-cardiac surgery OR gynecologic oncology OR gynecologic cancer)
**Scopus**	TITLE-ABS-KEY(("postoperative care" OR "postoperative monitoring" OR "postoperative recovery") AND ("critical care" OR "intensive care" OR ICU OR "high-dependency unit" OR HDU OR "intermediate care" OR "post-anesthesia care unit" OR PACU OR "advanced recovery room") AND ("major abdominal surgery" OR "abdominal surgery" OR "noncardiac surgery" OR "non-cardiac surgery" OR "gynecologic oncology" OR "gynecologic cancer"))
**CENTRAL**	("postoperative care" OR "postoperative monitoring" OR "postoperative recovery") AND ("critical care" OR "intensive care" OR ICU OR "high dependency unit" OR HDU OR "intermediate care" OR "post-anesthesia care unit" OR PACU OR "advanced recovery room") AND ("abdominal surgery" OR "non-cardiac surgery" OR "gynecologic oncology" OR "gynecologic cancer")
**Google Scholar**	"postoperative" ("critical care" OR "intensive care" OR ICU OR "high dependency" OR PACU OR "advanced recovery room") ("abdominal surgery" OR "non-cardiac surgery" OR "gynecologic oncology")
**ClinicalTrials.gov**	Condition/disease and other terms combining: postoperative care OR postoperative monitoring AND critical care OR intensive care OR ICU OR high-dependency OR PACU OR advanced recovery AND abdominal surgery OR non-cardiac surgery OR gynecologic oncology

Item	Critical appraisal questions for cohort studies
Q1	Were the two groups similar and recruited from the same population?
Q2	Were the exposures measured similarly to assign people to both exposed and unexposed groups?
Q3	Was the exposure measured in a valid and reliable way?
Q4	Were confounding factors identified?
Q5	Were strategies to deal with confounding factors stated?
Q6	Were the groups/participants free of the outcome at the start of the study or at the moment of exposure?
Q7	Were the outcomes measured in a valid and reliable way?
Q8	Was the follow-up time reported and sufficient for the outcomes to occur?
Q9	Was follow-up complete and, if not, were reasons for loss to follow-up described and explored?
Q10	Were strategies to address incomplete follow-up utilized?
Q11	Was appropriate statistical analysis used?
**Item**	**Critical appraisal question for quasi-experimental studies**
Q1	Is it clear in the study what is the “cause” and what is the “effect”?
Q2	Were the participants included in any comparisons similar?
Q3	Were participants included in any comparisons receiving similar treatment/care, other than the exposure or intervention of interest?
Q4	Was there a control group?
Q5	Were there multiple measurements of the outcome both before and after the intervention/exposure?
Q6	Was follow-up complete and, if not, were differences between groups in follow-up adequately described and analyzed?
Q7	Were the outcomes of participants included in any comparisons measured in the same way?
Q8	Were outcomes measured in a reliable way?
Q9	Was appropriate statistical analysis used?
**Item**	**Critical appraisal questions for case series**
Q1	Were there clear criteria for inclusion in the case series?
Q2	Was the condition measured in a standard, reliable way for all participants included in the case series?

**Q3**	Were valid methods used for identification of the condition for all participants included in the case series?
**Q4**	Did the case series have consecutive inclusion of participants?
**Q5**	Did the case series have complete inclusion of participants?
**Q6**	Was there clear reporting of the demographics of the participants in the study?
**Q7**	Was there clear reporting of clinical information of the participants?
**Q8**	Were the outcomes or follow-up results of cases clearly reported?
**Q9**	Was there clear reporting of the presenting site(s)/clinic(s) demographic information?
**Q10**	Was statistical analysis appropriate?

Table S1 (expanded). Characteristics of included studies								
**Study**	**Setting**	**Design / specialty**	**Eligibility / baseline risk**	**Pathway / intervention**	**Comparator / duration**	**Main outcomes / findings**	**Follow-up**	**Limitations / JBI**
**Koning, 2024**	Netherlands; teaching hospital	Interrupted time-series; mixed non-cardiac surgery	Selected intermediate/high-risk procedures	Planned PACU; individualized monitored care	IMCU; 20 h vs 6 h	LOS: adjusted GMR 0.77 (95% CI 0.66–0.91); complications 8.5% vs 9.6%; ICU admission 4.0% vs 4.1%	1 y	Retrospective, single center, secular confounding; **some concerns**
**Harmse, 2023**	South Africa; tertiary hospital	Retrospective audit; mixed surgery	High-risk patients; ASA, RCRI, frailty, STOP-Bang, NSQIP considered	Planned PAHCU; anesthesiologist-led allocation	None; usually <24 h	ICU transfer 1.07%; mortality 0.09%; predictors of >24-h stay assessed	In-hospital	No comparator; clinician selection; COVID-19 effects; **descriptive evidence**
**Ludbrook, 2023**	Australia; tertiary hospital	Prospective comparative; mixed non-cardiac surgery	NSQIP mortality risk 0.7–5%	Planned ARRC; protocolized enhanced monitoring	Usual PACU/ward; ≤24 h	Days at home +1.74 d (95% CI 0.11–3.36); fewer later MER events	90 d	Non-randomized; residual confounding; underpowered secondary outcomes;

								some concerns
**Costa-Pinto, 2023**	Australia; teaching hospital	Retrospective cohort; mixed high-risk surgery	Patients admitted to recovery HDU	Planned recovery HDU; anesthesia/ICU co-management	None; median 12.6 h	ICU admission 13.0%; MET call 6.4%; mortality 2.9%; LOS 8.3 d	In-hospital	No comparator; retrospective, single center; descriptive evidence
**Ludbrook, 2021**	Australia; multicenter hospitals	Before-after feasibility; mixed surgery	Predicted mortality approximately 1–4%	Planned ARRC; structured monitoring	Pre-ARRC usual care; ≤24 h	Feasibility, MER-level events, mortality and resource use	90 d	Small, non-randomized feasibility study; center heterogeneity; moderate concerns
**Nelson, 2020**	USA; academic hospital	Retrospective cohort; mixed surgery	Adult PACU patients; risk-adjusted using Vizient models	Capacity-driven overnight PACU	No overnight PACU; ≤24 h	Increased resource LOS and hospital costs; mortality assessed	In-hospital	Non-random allocation; bed-availability confounding; some concerns
**van Tunen, 2020**	Netherlands; university hospital	Retrospective cohort; mixed	Protocol-based PACU indications;	Mixed planned/unplann	Internal pathway	Correct planning 71%; 30-d	30 d	Heterogeneous groups; small subgroups;

		elective surgery	emergency cases excluded	ed PACU pathways	groups; <24 h	readmission/CPR/mortality assessed		confounding by indication; **substantial concerns**
**Ganter, 2014**	Switzerland; city hospital	Prospective audit; mixed surgery	Consecutive PACU patients	PACU / IMC-level postoperative care	None; 3.2 vs 15.1 h	Pain 27%→13%; PONV 3%; predictors of prolonged PACU stay	PACU stay	No comparator; heterogeneous population; no longer-term follow-up; **descriptive evidence**
**Heller, 2008**	UK; general hospital	Before-after service evaluation; major elective surgery	Patients requiring level-2 postoperative care	Planned POSU; anesthetic oversight, nurse-led discharge	Pre-POSU era; ~24 h	Increased elective surgical throughput; reduced cancellations; 30-d mortality 2%	30 d	No formal effectiveness framework or adjusted analysis; **substantial concerns**
